# Evidence for a wide extra-astrocytic distribution of S100B in human brain

**DOI:** 10.1186/1471-2202-8-2

**Published:** 2007-01-02

**Authors:** Johann Steiner, Hans-Gert Bernstein, Hendrik Bielau, Annika Berndt, Ralf Brisch, Christian Mawrin, Gerburg Keilhoff, Bernhard Bogerts

**Affiliations:** 1 Department of Psychiatry, University of Magdeburg, Leipziger Str. 44, D-39120 Magdeburg, Germany; 2 Institute of Neuropathology, University of Jena, Erlanger Allee 101, D-07743 Jena, Germany; 3 Institute of Medical Neurobiology, University of Magdeburg, Leipziger Str. 44, D-39120 Magdeburg, Germany

## Abstract

**Background:**

S100B is considered an astrocytic in-situ marker and protein levels in cerebrospinal fluid (CSF) or serum are often used as biomarker for astrocytic damage or dysfunction. However, studies on S100B in the human brain are rare. Thus, the distribution of S100B was studied by immunohistochemistry in adult human brains to evaluate its cell-type specificity.

**Results:**

Contrary to glial fibrillary acidic protein (GFAP), which selectively labels astrocytes and shows only faint ependymal immunopositivity, a less uniform staining pattern was seen in the case of S100B. Cells with astrocytic morphology were primarily stained by S100B in the human cortex, while only 20% (14–30%) or 14% (7–35%) of all immunopositive cells showed oligodendrocytic morphology in the dorsolateral prefrontal and temporal cortices, respectively. In the white matter, however, most immunostained cells resembled oligodendrocytes [frontal: 75% (57–85%); temporal: 73% (59–87%); parietal: 79% (62–89%); corpus callosum: 93% (86–97%)]. S100B was also found in ependymal cells, the choroid plexus epithelium, vascular endothelial cells, lymphocytes, and several neurones. Anti-myelin basic protein (MBP) immunolabelling showed an association of S100B with myelinated fibres, whereas GFAP double staining revealed a distinct subpopulation of cells with astrocytic morphology, which solely expressed S100B but not GFAP. Some of these cells showed co-localization of S100B and A2B5 and may be characterized as O2A glial progenitor cells. However, S100B was not detected in microglial cells, as revealed by double-immunolabelling with HLA-DR.

**Conclusion:**

S100B is localized in many neural cell-types and is less astrocyte-specific than GFAP. These are important results in order to avoid misinterpretation in the identification of normal and pathological cell types in situ and in clinical studies since S100B is continuously used as an astrocytic marker in animal models and various human diseases.

## 1. Background

S100B protein is a Ca2+, Cu2+ and Zn2+ binding member of the S100-calmodulin-troponin superfamily and is primarily found in high abundance within the nervous system [1].

21 kDa homodimers are the predominant form in which the protein exists within cells. A number of intracellular growth-associated target proteins have been revealed, such as growth-associated protein 43 (GAP-43), the regulatory domain of protein kinase C (PKC), the anti-apoptotic factor Bcl-2 or the tumour suppressor protein P53 [2]. S100B has also been found to regulate protein ubiquitination via interaction with Sgt1 [3] and regulate the assembly of cytoskeleton components such as microtubules, glial fibrillary acidic protein (GFAP) or vimentin [4].

However, S100B is not only implicated in the regulation of intracellular processes, but, it is also a secretory protein and exhibits cytokine-like activities, which mediate the interactions among glial cells and between glial cells and neurones. These effects are induced, in part, by interaction of S100B with the receptor for advanced glycation end products (RAGE), a multiligand receptor that has been shown to transduce inflammatory stimuli and effects of several neurotrophic and neurotoxic factors [2]. Secretion of S100B from astrocytes is stimulated under metabolic stress (oxygen, serum and glucose deprivation) and is suppressed by glutamate [5,6]. S100B acts in a dose-dependent manner: Nanomolar levels stimulate neurite growth and promote neurone survival. However, micromolar levels result in opposite effects and can even induce neuronal apoptosis, leading to the induction of pro-inflammatory cytokines such as interleukin1β (IL-1β) or tumour necrosis factor α (TNF-α), and inflammatory stress-related enzymes such as inducible nitric oxide synthase (iNOS) [7,8].

High levels of S100B in cerebrospinal fluid (CSF) and peripheral blood have been observed in clinical research on Alzheimer's disease, stroke, traumatic brain injury, meningoencephalitis, mood disorders and schizophrenia [9-13]. Elevated levels of S100B have been increasingly considered a biomarker for astrocytic damage or dysfunction in this context. This may have arisen from several postmortem studies on Alzheimer's disease and Down's syndrome describing predominant S100B immunostaining in reactive astrocytes surrounding neuritic plaques [14-16]. Other studies on the cell-type specificity of S100B in human brain are rare [17,18]. The cell-type specific localization of S100B in white matter has been minimally studied in human brain. However, higher contents of S100 have been found in white matter homogenates relative to cortex homogenates by the discoverer of the protein family [19].

In addition, it is sometimes overlooked that S100B containing cell types have also been identified outside the central nervous system and there are physiological stimuli for a release of S100B into the serum or CSF, which are not related to central nervous system diseases. Examples are physical exercise [20], stress [21,22], fasting [23], critically ill patients [24], cardiac arrest [25] and extracranial injuries [26,27] without brain injury.

The aim of the present work was to analyze the immunohistochemical distribution of S100B in adult human brain. We investigated telencephalic and diencephalic regions, including white matter of 14 healthy adults, to evaluate the cell-type specificity of S100B.

## 2. Results

### Western blot analysis

**Figure 1:**
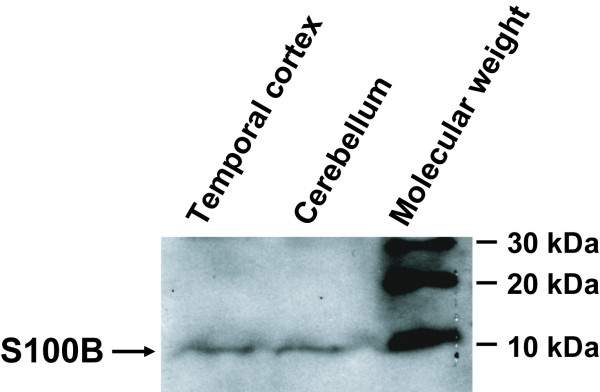
S100B immunoblot (DAKO, Glostrup, Denmark, dilution 1:500) indicating a single band of S100B protein in human brain homogenates.

Using the S100B antibody obtained from DAKO, we observed a single strong band with a molecular mass of about 10 kDa, representing the authentic molecule, in human adult brain (Figure 1). This is in accordance with the immunoblotting analysis performed by the manufacturer of the antibody (DAKO, Glostrup, Denmark, see data sheet) using recombinant S100A1, S100A2, S100A4 and S100A6, where no cross-reaction with other S100 proteins was observed.

### Preabsorption

No specific immunostaining was observed after preabsorption of the S100B antibody with the recombinant human-S100B protein.

### Immunohistochemistry

**Figure 2:**
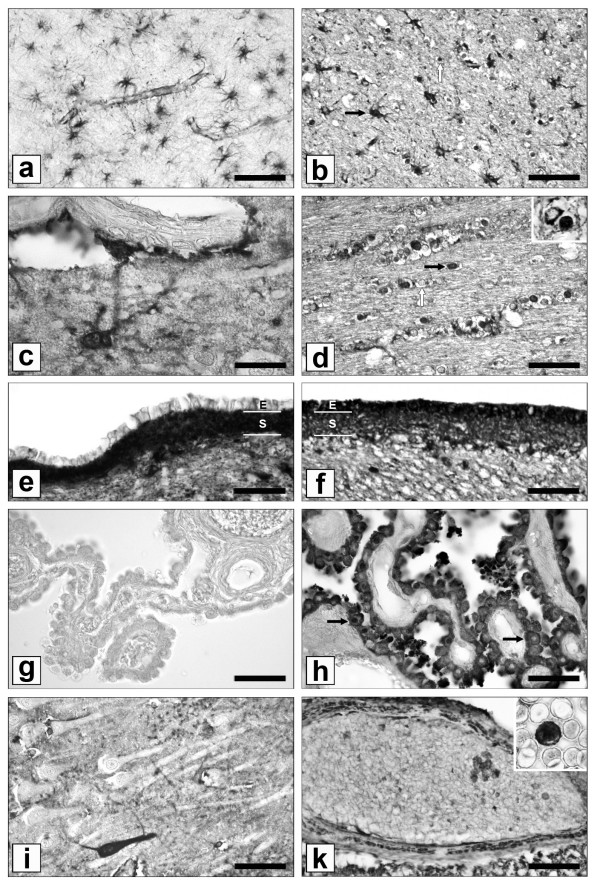
**GFAP- and S100B-single immunostaining**. **(a) **GFAP immunohistochemistry of the border between temporal cortex and adjacent white matter selectively labels star-shaped astrocytes and their processes. Blood vessels appear to be GFAP-positive because of their close association with pericapillary endfeet. **(b) **In contrast, various glial cell types are expressing S100B in this brain region, such as star-shaped cells with astrocytic morphology (black arrow) and spheroid cells with oligodendrocytic morphology (white arrow). **(c) **S100B stained astrocytic end feet (arrow: glial perivascular membrane) surround vascular endothelial cells. **(d) **"Pearl necklet" alignment of S100B positive (black arrow) and negative (white arrow) cells with oligodendrocytic morphology are associated with fibre tracts of the corpus callosum. Cytoplasmic and nuclear distribution of S100B is shown in the detail-picture. **(e) **The ependymal layer [E] around the lateral ventricle shows only weak GFAP staining, whereas the subependymal layer [S] shows intense staining of astrocytes and astrocytic processes. **(f) **Strongly S100B immunopositive ependymal [E] and subependymal [S] layers around the lateral ventricle. **(g) **Ependymal cells of the choroid plexus do not express GFAP but are heavily stained for S100B **(h)**; some S100B-positive cells display apical secrete granules; arrows indicate cells with cytoplasmic versus cytoplasmic and nuclear S100B expression. **(i) **S100B immunopositive CA1 hippocampal neurone. Neurones are rarely S100B positive. **(k) **S100B immunopositive lymphocytes and vascular smooth muscle cells. The detail picture shows a S100B stained lymphocyte from a blood smear. Bars: **a, b **= 100 μm; **d, e, f, g, h, I, k **= 50 μm; **c, d (detail), k (detail) **= 20 μm.

The differential S100B- and GFAP-immunostaining pattern is summarized in Table 1 and 2. GFAP immunohistochemistry selectively labelled astrocytes and their processes (Figure 2a), showed only faint ependymal immunopositivity and was negative in the choroid plexus epithelium (Figure 2e, g). In contrary, a less uniform staining pattern was seen in the case of S100B. Cells with astrocytic morphology in the human cortex primarily stained positive for S100B, while only 20% (14–30%) or 14% (7–35%) of all immunopositive cells showed oligodendrocytic morphology in the dorsolateral prefrontal and temporal cortex, respectively. In the white matter, however, most immunostained cells resembled oligodendrocytes [frontal: 75% (57–85%); temporal: 73% (59–87%); parietal: 79% (62–89%); corpus callosum: 93% (86–97%); Figure 2b, d]. Both GFAP- and S100B-immunostaining were seen in the subependymal layer and the perivascular (glia limitans) compartment and the perivascular membrane formed by astrocytic endfeet (Figure 2c, e, f). Of note, we observed strong S100B immunopositivity of the ependymal and choroids plexus epithelia (Figure 2f, h). In addition, S100B was also found in a few neurones and a subpopulation of lymphocytes (Figure 2i, k). By contrast, GFAP expression was detected neither in neurones nor in lymphocytes by the monoclonal antibody. The intracellular localization also varied: S100B was found in the nucleus and cytoplasm whereas GFAP was confined to the cytoplasm.

### Double immunolabelling and immunofluorescence staining

**Figure 3:**
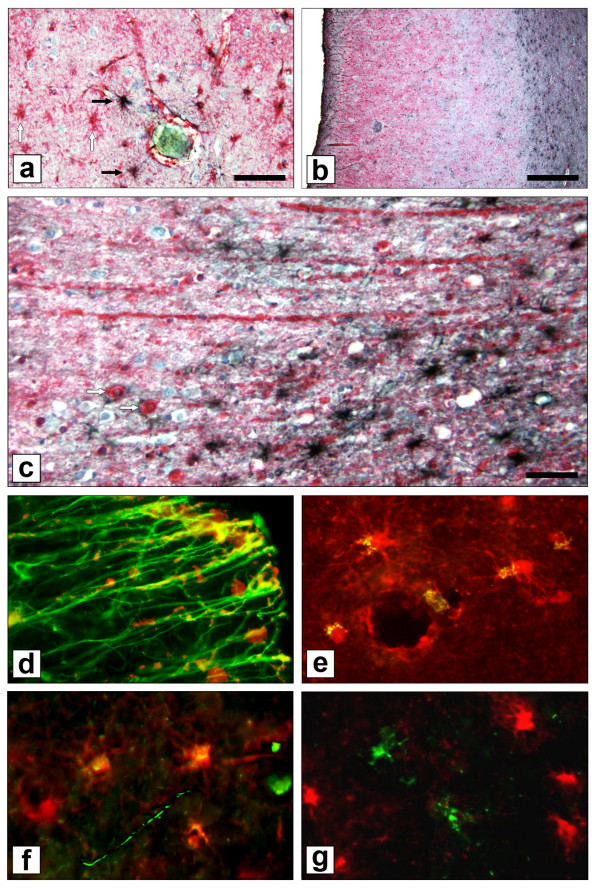
**Double immunostaining and immunofluorescence staining**. **(a) **S100B (red)/GFAP (black) double immunolabelling with haematoxylin-counterstaining (blue) revealed distinct populations of cells with astrocytic morphology in the temporal cortex. White arrows indicate S100B single positive cells with astrocytic morphology; black arrows indicate GFAP immunopositive cells with astrocytic morphology. **(b) **Temporal brain region (overview): The majority of GFAP immunopositive (black) astrocytes were found in the glial limitans and perivascular membrane or in the adjacent white matter. Less GFAP immunopositive cells were observed in the cortex. **(c) **Temporal cortex and adjacent white matter (higher magnification): S100B positive (red) radial fibres and oligodendrocytes (white arrows) are shown in comparison to GFAP positive (black) astrocytes; haematoxylin-counterstaining (blue). **(d) **Co-localization (yellow) of S100B (red) and MBP-positive (green) myelinated fibres, extending from the temporal cortex to the adjacent white matter. **(e) **Co-localization (yellow) of S100B (red) and MOG-positive (green) oligodendrocytes. **(f) **Co-localization (yellow) of S100B (red) and A2B5-immunopositive (green) O2A glial progenitor cells in the temporal cortex. **(g) **No co-localization (yellow) was observed for S100B (red) and microglial HLA-DR (green). Bars: **a **= 100 μm; **b **= 500 μm; **c **= 30 μm.

Double immunolabelling revealed a distinct subpopulation of cells with astrocytic morphology, which were either positive for S100B or GFAP, or expressed both antigens (Figure 3a, b). Some of these were A2B5-immunopositive O2A glial progenitor cells (Figure 3f). S100B-positive fibres were also observed, which correspond to myelinated fibers as shown by double immunolabelling for S100B and MBP (Figure 3c, d). Oligodendrocytic S100B expression was confirmed by co-localization with MOG (Figure 3e). However, no microglial S100B immunolabelling was observed by HLA-DR double immunostaining (Figure 3g).

## 3. Discussion and Conclusion

The cell-type specific localization of S100B in the brain has already been investigated in various species, including mouse, rat, cat, and lizard [28-33]. These studies found S100B in all glial cells, including astrocytes, ependymal cells, oligodendrocytes, microglial and Schwann cells. Studies on the cell-type specific expression of S100B in human brain are rare, but astrocytic and oligodendrocytic expression have been observed [23,47]. Immunohistochemical postmortem studies on S100B in human temporal cortex in Alzheimer's disease and Down's syndrome may have led to the assumption that S100B is an astrocyte-specific marker (see introduction) [13,28,44]. This is in accordance with our findings on grey matter, indicating a predominantly astrocytic S100B expression pattern in the dorsolateral prefrontal and temporal cortex of individuals without neurological or psychiatric disorders. However the majority of S100B immunopositive cells in the white matter resembled oligodendrocytes, which is in accordance with an early publication by Moore et al. (the discoverer of S100) who observed a higher content of S100 in white matter homogenates relative to cortical homogenates [19]. It has been demonstrated in rodents that S100B is primarily confined to immature oligodendrocytes, which may be activated in the context of a regenerative myelination processes [28,31,34,35]. Deloulme et al. [28] detected S100B protein in up to 40% of MBP-positive oligodendrocytes in the corpus callosum of mice and Rickmann et al. [34] found an association of S100B with paranodal loops and outer mesaxons of myelin sheaths in rats by electron microscopic immunohistochemistry.

Microglial staining has only been detected in the rat by Adami et al. so far, but has not been observed in our present study on human brain [30]. Our study is the first reporting of a small subpopulation of S100B containing neurones in human brain tissue. This is in accordance with findings in the mouse [29,36] and rat [33,37], but is in contrary to Li et al., who was unable to detect S100B immunopositive neurones in adult human brain [18]. Using a different antibody (mouse monoclonal anti-S100B from IBL, Minnesota) and the absence of antigen demasking techniques in the study by Li et al. might explain these different observations, as the importance of antigen retrieval methods to detect S100B immunopositive neurones has already been addressed by Yang et al. in mice [37].

Interestingly, the ependyma and the choroid plexus epithelia were strongly S100B immunopositive in our study as previously noted in the rat [38]. The surface ependyma is known to express epithelial markers and faint GFAP-staining. It is considered to be developmentally related to astrocytes, while the choroid plexus epithelium is a specialized ependyma, which differs from the surface ependyma: vimentin, cytokeratin and S100B continue to be expressed, but GFAP is not expressed [39]. Thus, the observed distribution pattern of S100B in the present study on human brain completely matches that reported in mouse and rat, indicating that cellular function might be very similar among mammalians.

Our study reveals a subpopulation of cells with astrocytic morphology, which were only immunopositive for S100B but not for GFAP. One explanation might be that some cells with astrocytic morphology were S100B- and A2B5-immunopositive O2A glial progenitor cells, which do not express GFAP [40].

In conclusion, S100B is a less astrocyte-specific marker than GFAP. Therefore, it should not be generally assumed that astrocytic damage or dysfunction is the main source for increased S100B concentrations in the case of studies with plasma, serum or cerebrospinal fluid. S100B may also be released during non-astrocyte cell death as it is also localized in O2A glial progenitor cells, oligodendrocytes, ependymal and plexus epithelial cells and neurones. In addition, S100B protein has also been noted outside the central nervous system. Immunocytochemical studies detected S100B in 5–25% of CD8+ peripheral blood T cells [41], in adipose tissue, melanocytes, injured myocardium, satellite cells of dorsal root ganglia and Schwann-cells of the peripheral nervous system [2]. There is little information about the functional implications, but all these cell types are potential sources for S100B in serum.

In contrast, only minor sources of GFAP have been detected outside the central nervous system. Traces of GFAP have been found in the peripheral nervous system, macrophages, lymphocytes, lens epithelial cells, and few human breast myoepithelial cells [42-45]. Moreover, the peripheral GFAP is a shorter protein version and may be antigenically and structurally different, as *monoclonal anti-brain *GFAP antibodies have failed to recognize peripheral GFAP [46,47].

In summary, serum and CSF S100B, different from GFAP, has many putative cellular sources. This is important to avoid misinterpretation of normal and pathological cell type identifications in situ and in clinical studies since S100B is continuously used as an astrocytic marker in animal models and various human diseases.

## 4. Methods

### Human brain tissue for Western blot analysis and immunofluorescence staining

The brain of a 32 year old woman (who died from acute myocarditis associated with lupus) was removed from the cranium within 20 hrs after death, dissected into small pieces, snap-frozen in liquid nitrogen and stored.

### Human brain tissue for immunohistochemistry

Postmortem brain tissue of 14 individuals without neurological or psychiatric disorders was used for the present study (mean age = 58.4 years; 7 males, 7 females). All brains were obtained from the Magdeburg Brain Collection. The collection of the human brain material was done in accordance with German laws and the rules outlined by the local ethics committee. Brains were removed within 48 hrs after death. The tissue preparation was performed as previously described [48]. Briefly, brains were fixed in toto in 8% phosphate-buffered formaldehyde (pH 7.0) for 3 months. After embedding the brains in paraplast, serial coronal 20 μm thick whole brain sections were cut on a microtome and mounted. For immunohistochemical staining, sections about 1 cm rostral to the genu of the corpus callosum and sections containing the hippocampus, as well as thalamus and lateral ventricles at the level of the mamillary bodies were selected.

*Blood smears *from two healthy subjects were stained for S100B for closer identification of S100B immunopositive white blood cells, which were found in the vessels of brain sections. The blood smears were fixed in methanol for 1 h.

### Western blot analysis

Frozen tissue samples from the temporal cortex and cerebellum was homogenized in lysis buffer [10 mM Tris (pH 7.4), 150 mM NaCl, 50 mM NaF, 1 mM EDTA, 1% Triton X-100, 0.1% SDS, 0.5% deoxycholat, 2.5 mM sodium pyrophosphate, 1 mM orthovanadate, 1 mM phenylmethylsulfonyl fluoride, 1 μg/ml leupeptin, and 10 μg/ml aprotinin, 1 mM DTT], and incubated on ice for 30 minutes. After centrifugation at 13,000 rpm for 15 minutes, the supernatant was collected to measure the total protein content. Protein concentration was measured by Bradford assay, with BSA as standard. Twenty micrograms of protein were loaded on 10% SDS-polyacrylamide gels for electrophoresis. After separation, proteins were transferred to a nitrocellulose membrane (Hybond C, Amersham Pharmacia Biotech, Freiburg, Germany) at 150 mA for 2 hours. Western blot analysis was done after blocking the membrane with 5% skim milk in TBST buffer for 1 hour. The membrane was incubated with the specific S100B antibody at 4°C overnight (DAKO, Glostrup, Denmark, dilution 1:500). The membrane was washed four times in TBST buffer. Secondary detection was performed using horseradish peroxidase-conjugated anti-rabbit immunoglobulin G (1:2,000; Amersham Pharmacia Biotech). After four washings with TBST, horseradish peroxidase activity was visualized by applying enhanced chemiluminescent substrate (Amersham Pharmacia Biotech) followed by exposure of the membrane to X-ray film.

### Immunohistochemistry – Single staining

Neighbouring sections were used for S100B or GFAP single-staining. Formalin-fixed tissue sections were deparaffinized and antigen demasking was performed by boiling the sections for 4 min in 10 mM citrate buffer (pH 6.0). Preincubation with 1.5% H_2_O_2 _for 10 min to block endogenous peroxidase activity was followed by blocking of nonspecific binding sites with 10% normal goat serum for 60 min and repeated washings with PBS. The primary antibody was diluted in PBS and applied for 48 h at 4°C (both antibodies were from DAKO, Glostrup, Denmark: polyclonal rabbit anti-recombinant human S100B, according to the manufacturer non cross-reactive with S100A1, S100A2, S100A4, and S100A6, dilution 1:50; monoclonal mouse anti-human brain glial fibrillary acidic protein (GFAP), dilution 1:100). Thereafter, sections were processed with the LSAB (labelled streptavidin biotin) method (DAKO, Glostrup, Denmark) and the reaction product was visualized with 3,3-diaminobenzidine. The colour reaction of the S100B staining was enhanced by adding 2 ml of a 0.5% (v/v) nickel ammonium sulphate solution to the diaminobenzidine [49]. *Preabsorption*: Specificity of the S100B immunoreaction was controlled by preabsorption of 2 ml antibody working dilution with 0.8 mg recombinant human-S100B (Biotrend, Cologne, Germany) for 24 h. No specific staining was observed after preabsorption with the protein. *Double staining*: Anti S100B primary antibody was applied for 48 h at 4°C. Thereafter, sections were incubated with EnVision Labelled Polymer (alkaline phosphatase-labelled anti-mouse and anti-rabbit antibodies, DAKO, Glostrup, Denmark) and the reaction product was visualized with Liquid permanent red (DAKO, Glostrup, Denmark). Subsequent GFAP immunostaining was performed according to the single-staining protocol after antibody elution with 0.1 M glycine-HCl for 10 min (pH 2.0). Counterstaining was performed with 50% (v/v) Harris Haematoxylin Solution (Sigma, St. Louis, USA) for 30 s.

### Immunofluorescence staining

A tissue sample from the temporal cortex and adjacent white matter was fixed in 4% buffered paraformaldehyde (pH 7.4) at 4°C overnight, cryoprotected in a solution of 30% sucrose (Merck, Darmstadt, Germany) in 0.4% buffered paraformaldehyde (pH 7.4) for 2 days, and rapidly frozen at -20°C using 2-methylbutane (Roth, Karlsruhe, Germany). Serial sagittal 20 μm thick sections were cut on a cryostat (Jung Frigocut 2800 E, Leica, Bensheim, Germany).

Free-floating sections were washed and incubated with the corresponding antibodies: polyclonal rabbit anti-recombinant human S100B (DAKO, Glostrup, Denmark, dilution 1:50), monoclonal mouse anti-myelin basic protein (anti-MBP, Chemicon, Hampshire, United Kingdom, dilution 1:100), monoclonal mouse anti-myelin oligodendrocyte glycoprotein (anti-MOG, Chemicon, Hampshire, United Kingdom, dilution 1:500), monoclonal mouse anti-A2B5 (Chemicon, Hampshire, United Kingdom, dilution 1:100), or monoclonal mouse anti-human HLA-DR-alpha-chain (Clone TAL.1B5, DAKO, Glostrup, Denmark, dilution 1:1000) in PBS with 0.3% Triton X-100 and 1% normal goat serum overnight at 4°C. Following this, slices were washed in PBS (3 × 5 min) and incubated overnight with a combination of secondary antibodies (Molecular Probes, Göttingen, Germany, dilution 1:500): goat anti-rabbit-IgG Alexa Fluor 546 or goat anti-mouse-IgG Alexa Fluor 488. Sections were examined using a fluorescence microscope (Axiophot, Zeiss, Germany) equipped with fluorescein, rhodamine and DAPI optics. Control reactions (substitution of the primary antisera with PBS) yielded negative results, i.e. no specific immunostaining was seen in these sections.

#### Cytological classification

The *cytological classification *of immunopositive cells as neurones, astrocytes, oligodendrocytes or microglia was performed according to established cytomorphological criteria [50]: Neuronal nuclei are large, round with a single prominent nucleolus ("fried-egg" nuclei) and are surrounded by substantial cytoplasm. Astrocytes have less cytoplasm surrounding large, more or less rounded nuclei and exhibit many star-shaped processes. Fibrous astrocytes are found predominantly in the white matter; they possess numerous elongated processes. Protoplasmic astrocytes are present among collections of neuronal cell bodies (grey matter). Their processes are less numerous and considerably more swollen in appearance than those of fibrous astrocytes. Oligodendrocytes are associated with fibre tracts in typical "pearl necklet" alignment. They are small spheroid glial cells with round, lymphocyte-like nuclei, a small cytoplasmic rim and few processes (less than astrocytes).

## Abbreviations

CSF: cerebrospinal fluid

DLPF: dorsolateral prefrontal

GAP-43: growth-associated protein 43

GFAP: glial fibrillary acidic protein

IL-1β: interleukin1β

iNOS: inducible nitric oxide synthase

MBP: myelin basic protein

MOG: myelin oligodendrocyte glycoprotein

PKC: protein kinase C

RAGE: receptor for advanced glycation end products

S.D.: standard deviation

TNF-α: tumour necrosis factor α

## Authors' contributions

JS, HGB and BB conceived and designed the study. JS interpreted the results, performed the statistical analysis, created the figures and drafted the manuscript. AB and RB evaluated the slides. HB documented the results and contributed to the interpretation of results. CM performed the neuropathological examination and Western blot analysis. GK was responsible for the immunofluorescence staining. All authors read and approved the final manuscript.

## Figures and Tables

**Table 1:** Cell-type specific S100B- and GFAP-immunostaining pattern. *Annotation*: ∅ immunonegative, + faint immunostaining, ++ strong immunostaining

**Cell type**	**S100B**	**GFAP**
**Astrocytes**	++, cytoplasm/nuclei	++, cytoplasm and processes
**Oligodendrocytes**	++, cytoplasm/nuclei	∅
**Neurones**	very few +, cytoplasm/nuclei	∅
**Ependyma**	++, cytoplasm/nuclei	+, cytoplasm
**Choroid plexus epithelium**	++, cytoplasm/nuclei	∅
**Lymphocytes**	++, cytoplasm/nuclei	∅

**Table 2:** Quantification of S100B immunopositive cells with astrocytic *versus *oligodendrocytic morphology. *Annotation*: Values are given as mean ± S.D.; *DLPF *dorsolateral prefrontal

**Brain region**	**Oligodendrocytic S100B+ [cells/mm^3^]**	**Astrocytic S100B+ [cells/mm^3^]**	**Oligodendrocytic S100B+ cells [mean % of all S100B+ glial cells (min % – max %)]**
**DLPF cortex**	1.247 ± 672	5.066 ± 2.674	20 (14 – 30)
**DLPF white matter**	14.423 ± 7.061	4.462 ± 2.376	75 (57 – 85)
**Temporal cortex**	1.207 ± 860	7.457 ± 3.964	14 (7 – 35)
**Temporal white matter**	11.229 ± 5.085	3.919 ± 2.310	73 (59 – 87)
**Parietal white matter**	9.279 ± 4.074	2.346 ± 1.357	79 (62 – 89)
**Corpus callosum**	10.038 ± 4.533	674 ± 338	93 (86 – 97)
